# Predominant global glomerulosclerosis in patients of upper urinary tract urothelial carcinoma with pre-existing renal function impairment is a predictor of poor renal outcomes

**DOI:** 10.1186/s12885-019-5414-x

**Published:** 2019-04-08

**Authors:** Sheng-Wen Niu, Peir-In Liang, Ming-Yen Lin, Shih-Meng Yeh, Yen-Yi Zhen, Yu-Han Chang, Pin-Chia Huang, Chi-Chi Hung, I-Ching Kuo, Hugo You-Hsien Lin, Mei-Chuan Kuo, Wei-Ming Li, Chun-Nung Huang, Wen-Jeng Wu, Li-Tzong Chen, Yi-Wen Chiu, Shang-Jyh Hwang

**Affiliations:** 1 Graduate Institute of Clinical Medicine, College of Medicine, Kaohsiung Medical University, No. 100, Shih-Chuan 1st Road, Kaohsiung, 80708 Taiwan; 2 Division of Nephrology, Department of Internal Medicine, Kaohsiung Medical University Hospital, Kaohsiung Medical University, No. 100, Tzyou 1st Road, 80708 Kaohsiung, Taiwan; 3 Department of Pathology, Kaohsiung Medical University Hospital, Kaohsiung Medical University, Kaohsiung, Taiwan; 4 Department of Urology, Kaohsiung Medical University Hospital, Kaohsiung Medical University, Kaohsiung, Taiwan; 5 National Institute of Cancer Research, National Health Research Institutes, Tainan, Taiwan; 6 Faculty of Medicine, Kaohsiung Medical University, Kaohsiung, Taiwan; 7 Kaohsiung Municipal Ta-Tung Hospital, Kaohsiung Medical University, Kaohsiung, Taiwan; 8 Yozen clinic, Kaohsiung Medical University, Kaohsiung, Taiwan; 9 Master of Public Health Degree Program, College of Public Health, National Taiwan University, Taipei, Taiwan

**Keywords:** Upper urinary tract urothelial carcinoma, Renal cell carcinoma, Renal survival, Tubulointerstitial nephropathy, Glomerulosclerosis

## Abstract

**Background:**

Incidence of renal dysfunction and risks of progression to end-stage renal disease (ESRD) were reported higher in upper urinary tract urothelial carcinoma (UTUC) than in renal cell carcinoma (RCC) patients after unilateral nephrectomy.

**Methods:**

Totally 193 renal cancer patients, including 132 UTUC and 61 RCC, were studied to clarify whether the pathological changes of the kidney remnant removed from nephrectomy and the clinical factors might predict the risk of ESRD. Renal tubulointerstitial (TI) score and global glomerulosclerosis (GGS) rate were examined by one pathologist and two nephrologists independently under same histopathological criteria**.**

**Results:**

The glomerular filtration rates at the time of surgery were lower in UTUC than RCC groups (*p* < 0.001). Average GGS score and average TI rate were higher in UTUC than in RCC groups (*p* < 0.001; *p* < 0.001). Competitive risk factor analysis revealed that abnormal GGS rate not related to age, predominant in UTUC with pre-existing renal function impairment, was a histopathological predictor of poor renal outcomes (creatinine doubling or ESRD) within 5 years in UTUC patients.

**Conclusion:**

Pre-existing renal function and pathological change of kidney remnant in both UTUC and RCC have the value for prediction of renal outcomes.

**Electronic supplementary material:**

The online version of this article (10.1186/s12885-019-5414-x) contains supplementary material, which is available to authorized users.

## Background

The outcomes of renal cancers after surgical unilateral nephrectomy include patient survival and possibility of renal function deterioration to end-stage renal disease (ESRD). Pathology of renal cancers can be divided into renal cell carcinoma (RCC) [1], origin from renal tubules, and urothelial cell carcinoma of renal pelvis and /or proximal ureter, named as upper urinary tract urothelial carcinoma (UTUC) [2]. RCC is much more common than UTUC in western countries [3], and UTUC contributes only 5% to all urothelial carcinoma (UC) [4]. However, prevalence of UTUC is not low in Taiwan, accounting for approximately 30% of all UCs [5], and is significantly, even 100 times higher, in areas endemic with aristolochic acid nephropathy (AAN) than the non-endemic counterparts [6].

The status of renal function in UTUC patients after surgical intervention of unilateral nephrectomy could be of normal, or of various stages of chronic kidney disease (CKD), or progression to ESRD. Our previous study demonstrated that 10.7% of UTUC patients underwent dialysis treatment [7]. Pathological findings of AAN contained extensive chronic tubulointerstitial (TI) fibrosis but sparse of glomerulosclerosis [8], which also resulted in progression into ESRD. Furthermore, AAN was reported strongly associated with the development of UTUC [9], and high incidence of urothelial cancers (UTUC and bladder cancer) were noted in ESRD patients received either renal transplantation or dialysis [10].

Despite that the exact mechanism of AAN and UTUC was still not explored completely, we were interested in status of renal function in relation to the histopathological changes of the kidney remnant from nephrectomy in UTUC and RCC patients. Clinical factors and pathological parameters were analyzed for prediction of renal survival (creatinine doubling and ESRD) after unilateral nephrectomy in groups of UTUC and RCC patients.

## Methods

### Subject data

**Fig. 1:**
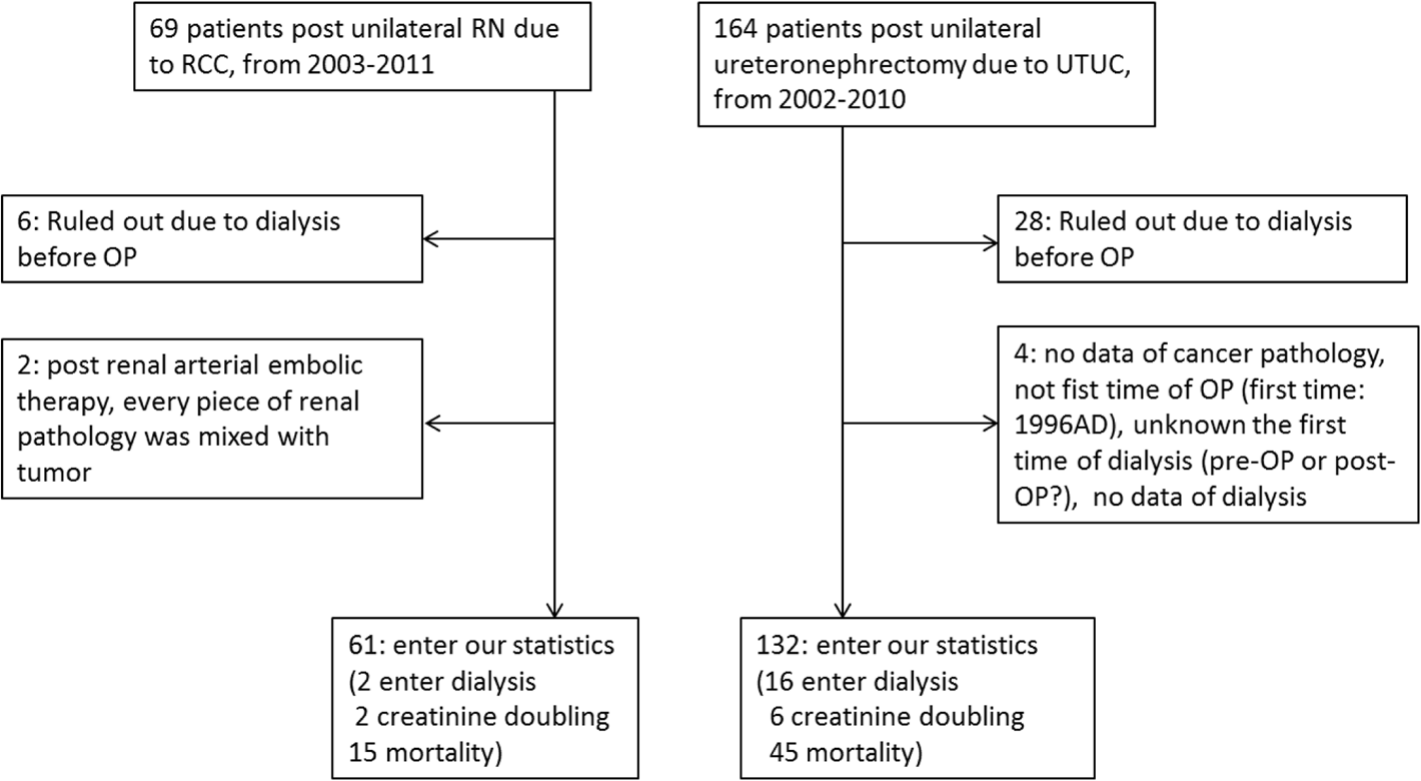
Flowchart of subject screening

We retrospectively analyzed data of 132 non-metastatic UTUC patients between 2002 and 2010, and 61 non-metastatic RCC patients between 2003 and 2011. All patients had undergone unilateral nephrectomy through either open or laparoscopic approach at our hospitals. Parameters of age, sex, smoking, Chinese herb use, and prevalence of hypertension, diabetes mellitus, hyperlipidemia, hydronephrosis, and kidney stones were recorded. We excluded subjects who had incomplete clinical information, received renal replacement therapy preoperatively, had no pathological evidence of UTUC, and had undergone surgery twice for UTUC. Location of tumor defined as either ureter or renal pelvis based on dominant tumor features, in a sequential order of the stage, grade, and size. The renal histopathological parameters were investigated by 3 specialists: 2 nephrologists and a pathologist. Moreover, the subjects were stratified into quartiles according to age (≤54, 55–64, 65–74, and ≥ 75 years) and sex for pre-existing CKD prevalence and other analyses. The flow chart is described in Fig. 1. The study protocol was approved by our Institutional Review Board (KMUH-IRB-20120138).

### Pre-existing CKD evaluation

To evaluate the patient’s pre-operation kidney function, the latest creatinine level obtained within 30 days preoperatively was collected. We used CKD Epidemiology Collaboration Equation (CKD-EPI) to calculate the estimated glomerular filtration rate (eGFR) [11].

$$ \mathrm{eGFR}=141\times \min\ {\left(\mathrm{Scr}/\upkappa, 1\right)}^{\upalpha}\times \max\ {\left(\mathrm{Scr}/\upkappa, 1\right)}^{\hbox{-} 1.209}\times 0{.993}^{\mathrm{Age}}\times 1.018\left(\mathrm{if}\ \mathrm{female}\right)\times 1.159\left(\mathrm{if}\mathrm{black}\right), $$

Were Scr is the serum creatinine, α is − 0.329 for women and − 0.411 for men, κ is 0.7 for women and 0.9 for men, min indicates the minimum Scr/κ or 1, and max indicates the maximum Scr/κ or 1. The pre-existing CKD stages of all patients were determined based on their pre-existing eGFR at the time of unilateral nephrectomy. All the patients were stratified into stages of CKD based on the Kidney Dialysis Outcomes Quality Initiative (K-DOKI) classification, as follows: stage 1, GFR > 90 mL/min/m^2^ with proteinuria or microalbuminuria; stage 2, GFR 60–89 mL/min/m^2^ with proteinuria or microalbuminuria; stage 3A, GFR = 45–59 mL/min/m^2^; stage 3B, GFR = 30–44 mL/min/m^2^; stage 4, GFR = 15–29 mL/min/m^2^; and stage 5, GFR < 15 mL/min/m^2^.

### Histochemical staining

Kidney specimen were thoroughly excision and representation, section are taken from the non-tumorous area, which is at least 1 cm distance from the tumor. Formalin-fixed paraffin embedded (FFPE) blocks of the non-tumorous kidney parenchyma were retrieved. Tissue sections of 3 μm were cut, deparaffinized, and rehydrated. Hematoxylin-eosin (H&E) stain, periodic acid-Schiff (PAS) stain and Masson trichrome stain were performed as recommended [12].

### Pathological evaluation

**Fig. 2:**
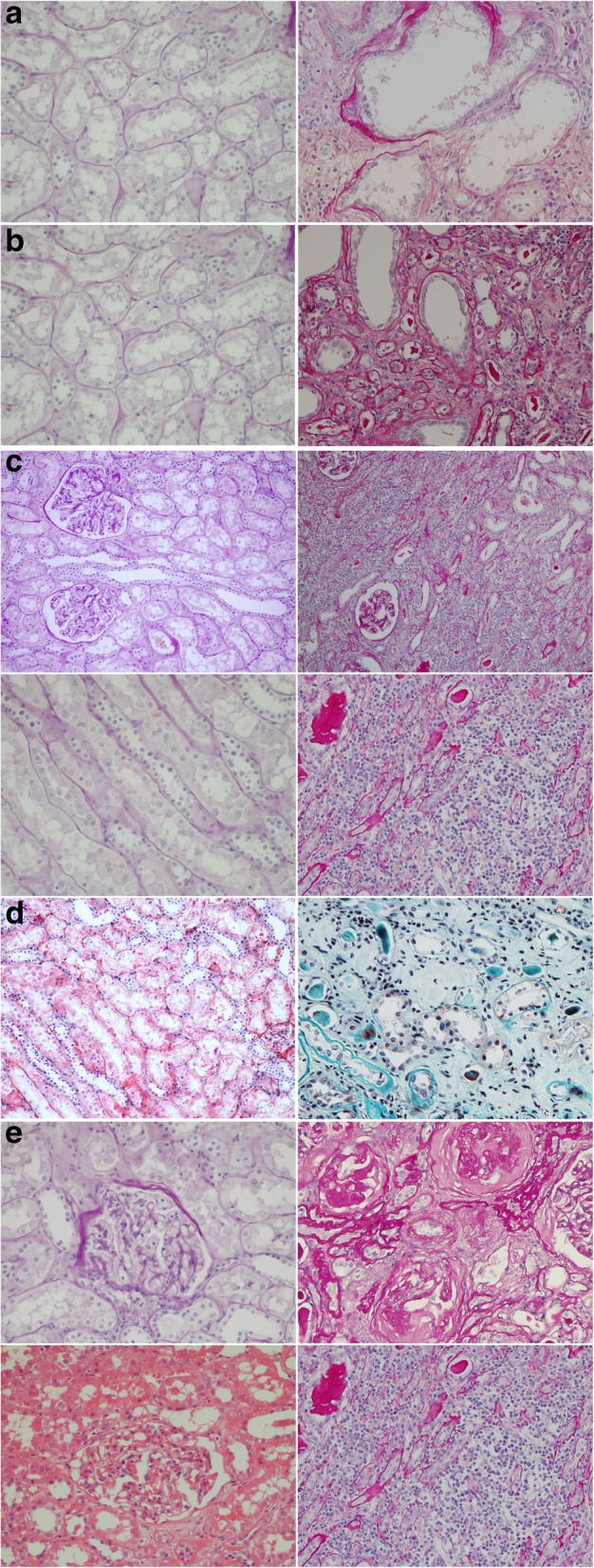
**a** Tubular necrosis. The left image shows a typical sample, and the right image shows tubular necrosis (vacuolated cells and sloughed, necrotic cells in tubular lumina, with some tubules lined by a flattened epithelium and some showing frank necrosis). (Periodic acid Schiff staining, × 20). **b** Tubular atrophy. The left image shows a typical sample, and the right image shows tubular atrophy (tubular basement membranes thickening and wrinkling, with simplified tubular epithelial cells, small round tubules with markedly flattened, uniform intratubular casts, and contraction of the tubular lumen adjacent to intact tubules). (Periodic acid Schiff staining, × 20). **c** Interstitial lymphocyte infiltration. Upper figures: The left image shows a typical sample, and the right image shows interstitial lymphocyte infiltration. The renal cortex shows a diffuse interstitial, predominantly mononuclear, inflammatory infiltrate with no changes in the glomerulus. (Periodic acid Schiff staining, × 10). Lower figures: The left image shows a typical sample, and the right image shows interstitial lymphocyte infiltration. Tubules in the center of the field are separated by inflammation and edema. (Periodic acid Schiff staining, × 20s). **d** Interstitial fibrosis. The left image shows a typical sample, and the right image shows interstitial fibrosis (connective tissue expansion through tubulointerstitial parenchyma and tubular loss). (Masson’s trichrome, × 10). **e** Global glomerulosclerosis score. The left figures show a typical sample, and the right figures shows GGS (a solidified nonretracted glomerular tuft with often recognizable tuft adhesions, splitting of Bowman’s capsule, and prominent periglomerular fibrosis). (Upper figures, periodic acid Schiff staining, × 20 and lower figures, H&E staining)

The global glomerulosclerosis (GGS) rate and tubulointerstitial (TI) score were semiquantified by 2 nephrologists and one pathologist, who were blinded to the patients’ clinical information. For cases with discrepancy, a consensus was made after review the slides together at a multi-headed microscope. The TI score was the sum of the severity level of four pathological features: tubular necrosis (Fig. 2a; 0: normal tubules, 1: rare single necrotic tubule, 2: several clusters of necrotic tubules, and 3: confluence of necrotic clusters), tubular atrophy (Fig. 2b; 0: normal tubules, 1: rare single atrophic tubule, 2: several clusters of atrophic tubules, and 3: confluence of atrophic tubular clusters), lymphocytic infiltrates (Fig. 2c; 0: absent, 1: few scattered cells, 2: group of lymphocytes, 3: and widespread infiltrates), and interstitial fibrosis (Fig. 2d; 0: absent, 1: minimal fibrosis, 2: moderate fibrosis, and 3: severe fibrosis), ranging from 0 to 12 [13]. GGS rate was the number of glomeruli with global glomerulosclerosis, defined as glomerulus with more than 50% of area involved by sclerosis, over the number of the glomeruli that can be found in the slides (Fig. 2e). Since GGS developed as an individual getting old, we compared the observed GGS rate with the estimated GGS, calculated by using an equation, (age X 0.5) – 10, that proposed by Smith et al. [14]. If the observed GGS rate exceeded the estimated GGS, it was considered as “abnormal GGS rate” (Table 3). For an example, a 40% of observed GGS in a 80 years old patient (estimated GGS is 80 × 0.5–10 = 30%) was considered abnormal.

### Postoperative follow-up

All the patients were followed by performing cystoscopic examination every 3 months in the first 2 years after nephrectomy, every 6 months in the next 2 years, and annually thereafter. During surveillance, physical examinations and cystoscopic, urine cytological, and periodic imaging studies were performed following institutional guidelines. Intraluminal recurrence was defined as the recurrence of tumors in the contralateral upper urinary tract or bladder. Metastatic progression was defined as tumor recurrence in the tumor bed or regional lymph nodes and distant metastasis.

### End points

The primary end point was renal outcomes, defined as creatinine doubling or dialysis. The secondary end point was all-cause mortality. If the patients died within 3 months of the primary end point, they were not defined as having the primary end point.

### Statistical analysis

Data were described as mean ± standard deviation (SD), frequency, or percentage. Student’s t test or one-way analysis of variance (ANOVA) was used for comparing the continuous variables between different groups and chi-squared test was used for comparing different distribution of categorical data. Multiple binary logistic regression was applied to explore factors associated with preexisting CKD-EPI and abnormal of GGS rate. Factors associated with TI score were evaluated by multiple linear regressions. We calculated follow-up time as time between the date of unilateral nephrectomy and the date of dialysis or creatinine doubling. The Kaplan-Meier method was used to estimate renal survival rates of the histological GGS normal and abnormal groups and test the difference between these two groups by log-rank test.

Because our patients were more likely to die than to reach renal outcomes, a competitive risk Fin-Gray regression model was used to identify independent associated predictors. All independent variables were included univariable analysis and selected into multivariable analysis under criteria of *p* < 0.1. All statistical analyses were performed using SPSS Version 19 (IBM, Armonk, NY, USA) or SAS 9.4 (SAS Institute Inc., Cary, NC,USA), and figures were made using GraphPad Prism 5.0 (GraphPad Software, Inc., California, USA). In all analyses, two-sided *p* < 0.05 was considered statistically significant.

## Results

### Differences of clinical, laboratory, and pathological characteristics between UTUC and RCC groups

**Fig. 3:**
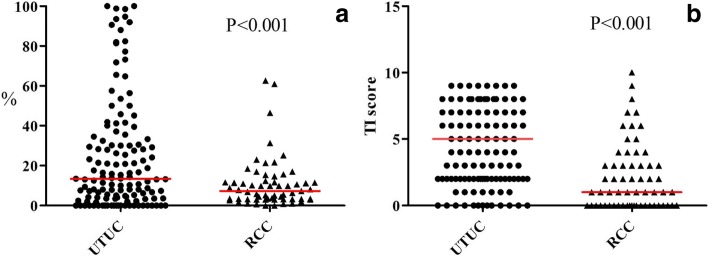
Distributions of global glomerulosclerosis (GGS) rates and tubulointerstitial (TI) scores. (**a**) GGS rate (**b**) TI score

**Fig. 4:**
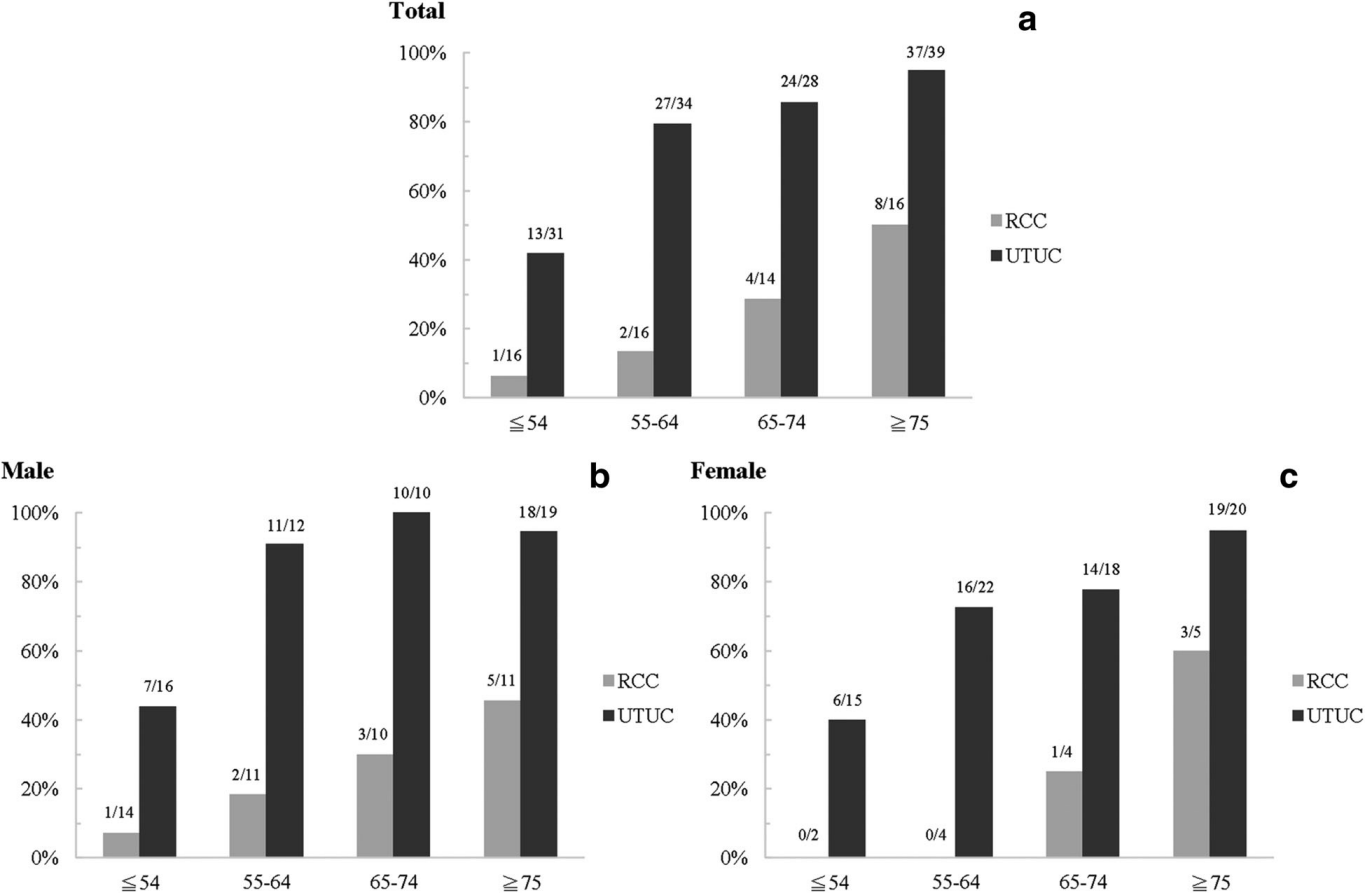
Distributions of pre-existing chronic kidney disease (CKD) in subjects with upper urinary tract urothelial carcinoma (UTUC) and renal cell carcinoma (RCC) by age and sex. **a** Overall (**b**) Male (**c**) Female

The clinical, laboratory, and pathological characteristics in UTUC and RCC groups are shown in Table 1. Compared to the RCC group, patients of UTUC group were significantly more women, older in age, more use of Chinese herb, and higher percentage of hydronephrosis. There were also significantly more pre-existing CKD, higher creatinine level, lower eGFR, anemic, increased risk of adverse renal outcome after surgery (*p* = 0.056), and higher overall mortality in UTUC group. Both the histopathological TI score and GGS score of kidney remnant shown in Fig. 3 were also significantly higher in the UTUC group. The average GGS rates were 24.12 ± 27.88% and 10.80 ± 12.60% in the UTUC and RCC groups, respectively (*p* < 0.001); and the average TI scores were 4.76 ± 2.92 and 2.13 ± 2.55 in the UTUC and RCC groups, respectively (*p* < 0.001). Distributions of pre-existing CKD in subjects with UTUC (*N* = 132) and RCC (*N* = 61), as stratified by age and sex, were shown in Fig. 4. The proportion of women in UTUC was higher (men vs women, 43.2% vs 56.8%) but prevalence of pre-existing CKD was slightly higher in UTUC men (80.7% vs 73.3%).

**Table 1:** Clinical, laboratory, and pathological characteristics of UTUC and RCC groups

Variable	UTUC	RCC	*p*
Female	75 (56.8%)	15 (24.6%)	< 0.001
Age (years)	67.9 ± 9.5	57.07 ± 11.58	< 0.001
Lifestyle			
Chinese herb	28 (25.0%)	4 (6.6%)	0.003
Smoking	27 (23.5%)	16 (26.2%)	0.686
Comorbidity			
Hypertension	50 (40.0%)	31 (51.7%)	0.134
Diabetes mellitus	29 (23.2%)	16 (26.7%)	0.607
Hyperlipidemia	8 (6.4%)	3 (5.0%)	0.706
Hydronephrosis	53 (47.3%)	2 (3.3%)	< 0.001
Pre-existing CKD	101 (76.5%)	15 (24.6%)	< 0.001
Renal stone	14 (12.7%)	4 (6.6%)	0.208
Laboratory data (Before nephrectomy)			
BUN (mg/dl)	21.77 ± 15.47	16.10 ± 10.94	0.015
Crea (mg/dl)	1.57 ± 1.30	1.14 ± 0.77	0.018
eGFR (ml/min/m^2^, CKD-EPI)	53.8 ± 24.7	80.0 ± 23.4	< 0.001
GPT (IU/L)	25.08 ± 24.84	30.79 ± 26.39	0.161
Albumin (mg/dl)	3.75 ± 0.55	4.04 ± 0.51	0.017
Na (mEq/L)	137.31 ± 4.29	139.10 ± 2.53	0.021
K (mEq/L)	3.96 ± 0.44	3.94 ± 0.39	0.698
WBC (× 1000/ul)	7.70 ± 3.21	7.02 ± 1.91	0.126
Hb (g/dL)	11.60 ± 2.07	13.40 ± 2.08	< 0.001
PLT (×1000/ul)	218.07 ± 76.62	229.30 ± 89.14	0.398
Renal histopathology			
Distribution of TI score			< 0.001*
0	13 (9.85%)	23 (37.70%)	
1~4	47 (35.61%)	28 (45.90%)	
5~8	64 (48.48%)	8 (13.12%)	
≧9	8 (6.06%)	2 (3.28%)	
Distribution of GGS rate			< 0.001*
0	24 (18.18%)	2 (3.28%)	
> 0~10	33 (25.00%)	36 (59.02%)	
> 10~25	29 (21.97%)	18 (29.51%)	
> 25~50	26 (19.70%)	3 (4.92%)	
> 50 (DGGS)	20 (15.15%)	2 (3.28%)	
Adverse renal outcome	22	4	0.056
Creatinine doubling	6	2	
Dialysis	16	2	
Overall mortality	45	15	0.019

*Abbreviations. UTUC* upper urinary tract urothelial carcinoma, *RCC* renal cell carcinoma, *CKD* chronic kidney disease, *GGS* global glomerulosclerosis, *TI* tubulointerstitial, *eGFR* glomerular filtration rates

Adverse renal outcomes, namely creatinine doubling and entering dialysis

Statistics done by unpaired t-test and *Chi-squared test; p less than 0.05 as significant

### Factors associated with pre-existing CKD in UTUC groups

Since there were patients with renal function impairment before unilateral nephrectomy and was more pronounced in UTUC group, to explore factors associated with pre-existing impaired renal function, results of logistic regression analysis of pre-existing CKD and all the clinical and pathological factors are shown in Table 2. Tumor type (UTUC), age, hydronephrosis, TI score, and abnormal GGS rate were independently associated with pre-existing CKD. After adjusting all possible confounding factors, presence of UTUC and age became the two factors with significantly higher odd ratio of pre-existing CKD. We further looked into UTUC group, and the pre-existing CKD was likely associated with age, however, presence of abnormal GGS rate became another significant factor (*p* = 0.049, Additional file 1: Table S1a) but not TI score. Since GGS could be a consequence of ageing or systemic diseases of hypertension or diabetes, multivariate analysis of logistic regression showed eGFR was the only factor significantly associated with abnormal GGS rate (*p* < 0.001, Additional file 1: Table S1b). The non-significances of age, hypertension, and diabetes excluded systemic factors responsible for abnormal GGS rate and suggested the possibility of unexplored mechanisms of GGS in UTUC patients.

**Table 2:** Factors associated with Pre-existing CKD by logistic regression

	COR	95% CI	*p* value	AOR	95%CI	*p* value
Tumor type						
RCC	1.00	Reference		1.00	Reference	
UTUC	9.99	(4.92,20.29)	< 0.001	3.09	(1.18,8.06)	0.021
Gender (male vs female)	0.65	(0.36,1.17)	0.149	–	–	–
Age	1.14	(1.10,1.19)	< 0.001	1.12	(1.07,1.18)	< 0.001
Chinese herb	1.39	(0.63,3.05)	0.418	–	–	–
Smoking	0.95	(0.47,1.89)	0.878	–	–	–
Hypertension	1.37	(0.75,2.49)	0.304	–	–	–
DM	1.13	(0.57,2.26)	0.727	–	–	–
Hyperlipidemia	1.18	(0.33,4.18)	0.800	–	–	–
Hydronephrosis	3.46	(1.68,7.10)	0.001	1.42	(0.56,3.63)	0.459
Renal stone	2.08	(0.71,6.12)	0.184	–	–	–
TI score	1.24	(1.12,1.38)	< 0.001	1.11	(0.96,1.28)	0.153
Abnormal GGS	4.79	(2.18,10.54)	< 0.001	2.68	(0.99,7.24)	0.052

*Abbreviation: OR* odd ratio, *CI* confidence interval, *COR* crude OR, *AOR* adjusted OR. Abbreviations are same as in Table 1

Despite of a higher TI score in UTUC group than in RCC group (Table 1), TI score was neither significantly associated with pre-existing CKD in overall patients (Table 2), nor a significant factor to pre-existing CKD and abnormal GGS in UTUC patients (Additional file 1: Table S1a, Table S1b).

### Factors associated with pre-existing CKD in RCC groups

Compared to the significant role of abnormal GGS rate to pre-existing CKD in UTUC group, there were just two cases of abnormal GGS rate in RCC patients. The too few the case numbers resulted in no significance associated with other factors (data not shown).

Though TI score was not a significant factor of pre-existing CKD in all patients as a whole (Table 2), in RCC group the pre-existing CKD significantly associated with TI score and age (Additional file 1: Table S2a, *p* = 0.010, *p* = 0.003), and TI score further significantly inversed associated with eGFR (*p* = 0.015, Additional file 1: Table S2b).

These observations might suggest that the pathogenesis of kidney function impairment in patients with UTUC and with RCC was different.

### Factors associated with the adverse renal progression

In overall 193 renal cancer patients, 22 of 132 (16.7%) UTUC patients and 4 of 61 (6.7%) RCC patients reached the primary end-point developed into ESRD received dialysis or showed creatinine doubling within 5 years after surgery. Competitive survival regression analysis after stepwise selection showed hypertension (*p* = 0.004), pre-existing CKD (*p* = 0.019), or abnormal GGS rate (*p* = 0.041) were risk factors associated with adverse renal outcomes (Table 3).

**Table 3:** Competing risk analysis in the UTUC and RCC groups to develop renal function impairment (creatinine doubling or receiving dialysis)

Variable	AHR (95%CI)	*p*
Hypertension (yes versus no)	4.12 (1.57, 10.84)	0.004
Pre-existing CKD (yes versus no)	6.20 (1.36, 28.30)	0.019
Abnormal GGS rate (yes versus no)	2.41 (1.04, 5.59)	0.041

Competing risk analysis was used as cause-specific hazard ratio estimation for renal outcomes, whereas death before dialysis or creatinine doubling or within 3 months after dialysis or creatinine doubling was considered as a competing event. Covariates in the multivariate model included tumor type (UTUC vs RCC), age, gender, a history of Chinese herb, smoking, diabetes, hyperlipidemia, hydronephrosis, renal stone, and TI score of renal histopathology. Abbreviations are the same as in Table 1

## Discussion

The present study demonstrate that UTUC patients had clinical features of female predominant, older in age, significantly higher risks of pre-existing CKD, and pathological findings of abnormal GGS rate and higher TI score than RCC ones. The pre-existing CKD in UTUC patients was associated with older age and abnormal GGS rate, and the abnormal GGS rate was associated with worse pre-existing GFR, but had no relation to age and systemic diseases. On the contrary, pre-existing CKD in RCC patients was associated with older age and higher TI score, and the higher TI score was related to worse pre-existing GFR. Patients with hypertension, pre-existing CKD before unilateral nephrectomy and abnormal GGS rates had a higher risk of creatinine doubling or developing ESRD within 5 years in not only UTUC but RCC patients.

Renal cancer is a common malignancy in countries with high socioeconomic development. Upper urinary tract urothelial carcinoma (UTUC) [2] and renal cell carcinoma (RCC) [1] are the two most commonly seen renal cancers. Although RCC are more common worldwide, especially in western countries, UTUC has higher incidence rate in certain regions, such as Balkan’s countries and Taiwan. Nephrectomy with or without ureterecctomy is for treatment of resectable renal cancer. Previous study shows that new-onset CKD developed in patient with T1a RCC after surgery [15]. Similar observation has been disclosed in UTUC patients [7]. In our study, we shows that hypertension, pre-existing CKD, and abnormal GGS rate are indicator for predicting kidney function deterioration after nephrectomy in both RCC and UTUC patients. Thus, it is likely that worse baseline kidney function plays a significant role in promoting CKD development in patient with renal cancer.

In nephrectomy specimens, non-neoplastic kidney disease frequently goes unrecognized [16]. Bijol et al. shows that patients with severe histopathologic findings predict a worse kidney outcome after radical nephrectomy [17]. In our study, we evaluate abnormal GGS rate and TI score in nephrectomy specimens of RCC and UTUC patients. We observed that, in contrast to RCC, UTUC patients had worse TI score and higher GGS rate. Abnormal GGS rate is strongly related to the pre-operation eGFR in UTUC patient, but it is a rare event in RCC patient. However, it is inverse in TI score, where higher TI score is associated with worse pre-operation eGFR in RCC patients but not UTUC. We concluded that the mechanism that lead to renal impairment in these two groups of cancer is likely different. In UTUC, it is the destruction of glomeruli that lead to renal impairment. This process is not related to other clinical factors, although age may play role in the process. Although tubulointerstitial injury did not show correlation with post-operation kidney function impairment, it is still a frequent event in UTUC. Unlike RCC, where tubulointerstitial injury is likely a consequence of glomerular destruction, this event is not associated with any factors that we look into in UTUC. Aristolochic acid (AA), a compound that is known to cause chronic kidney disease and urothelial carcinoma, has been purposed as a possible cause. Animal model shows that AA nephropathy (AAN) can cause severe tubulointerstitial injuries, including proximal tubular epithelial cell necrosis and transient acute kidney injury [18]. Chen et al. shows that AA related UTUC has higher incidence rate of ESRD than those non-AA related UTUC (28% vs 12%) [19]. However, AA exposure alone is not the sole explanation, because AAN usually shows extensive hypocellular interstitial sclerosis, tubular atrophy, and cellular atypia but spared glomeruli involvement [20]. Besides, we could not determine the incidence of AA intake nor AAN in our patients. The mechanism should be multifactorial and other possible pathogens are yet to be disclosed.

In Taiwan, the prevalent rate of CKD stage 3–5 is 6.9% [21]. In our cohort, the prevalent rate of pre-existing CKD in UTUC patients and RCC patients is 76.5 and 24.6%, respectively, and they are significantly higher than the general population in Taiwan. Abnormal GGS rate and TI score also associated with pre-existing CKD, although they are not independent predictors. These findings suggested that factors that accurately reflect the kidney reserved function are yet disclosed. Our study also shows that, in contrast with RCC, the UTUC are more correlated with pre-existing CKD. Although Hung et al. had reported that the aggressiveness of UTUC increased with the severity of CKD [22], our finding is the first to show that UTUC is an independent predictor for pre-existing CKD in renal cancer. Based on this observation, it is recommended to thoroughly evaluate the kidney function of patient with UTUC before surgical intervention.

The prevalence of CKD in women and men are variable worldwide [23]. In Taiwan, the incidences of male and female patients with CKD exhibiting renal progression are 11.64 and 12.52%, respectively [24]. In our study, the proportion of women in UTUC was higher (men vs women, 43.2% vs 56.8%) but the prevalence of pre-existing CKD was more commonly seen in men with UTUC (men vs women, 80.7% vs 73.3%). We hypothesize that although men have less likelihood of UTUC development, they are more susceptible to factors that injured the kidney. In animal model, male mice with orchiectomy may inhibit renal injury and female rat prevent renal injury through the expression of vascular endothelial growth factor and endothelial nitric oxide synthase [25, 26]. Previous studies also disclosed that estrogen has renoprotective effect and testosterone could enhance renal injury [27, 28]. Testosterone and 17β-estradiol have opposite effects on renal cells in female estrogen receptor knockout mice, as the former could aggravate podocyte apoptosis and glomerulosclerosis but the later inhibited the process [29]. Patients with AAN were usually combined with UTUC [30]. Taking AAN as a model, treatment with 17β-estradiol in male C57BL/6 mice with AAN reduced serum creatinine levels and attenuated renal proximal tubular injury and renal tubular epithelial cells apoptosis. In the mice kidney tissue and human renal proximal tubule cells (HK-2 cells), 17β-estradiol attenuated both AA-induced cell apoptosis via inhibiting the p53 signaling pathway [31]. Since most of our female patients are over 55 years old, and are likely menopause, we did not see the renoprotective effect in our cohort. But another recent study from our team revealed that Glycine N-methyltransferase attenuates AAN via decreasing NAD(P)H: quinone oxidoreductase 1 (NQO1) expression in female mouse hepatocytes, and this also implied: First, that male were less tolerant of AA toxicity than female, so acute kidney injury could warn male to stop AA-contained herbs intake [32], and continuous AA-contained herbs intake due to more tolerant of AA toxicity in female, may cause higher incidence of UTUC in Asia, especially in Taiwan [33]. Then, female were expected to have longer lifespan, this also contributed to higher incidence of UTUC in female.

Our study identified that both UTUC and RCC patients after unilateral nephrectomy with abnormal GGS rate, hypertension and pre-existing CKD have higher risk of creatinine doubling or dialysis within 5 years. Under this observation, we found some key points: First, UTUC patients had significantly higher risk of pre-existing CKD and higher TI scores than RCC ones, but UTUC patients with worse pre-existing GFR (EPI) have higher risk of abnormal GGS rates. Then, Both UTUC and RCC patients after unilateral nephrectomy with abnormal GGS rate have a higher risk of creatinine doubling or dialysis within 5 years, but there was no different risk of creatinine doubling or dialysis within 5 years between UTUC and RCC patients after unilateral nephrectomy. Finally, UTUC and RCC patients with hypertension had a lower 5-year postoperative renal survival. Hwang et al. reported that diabetes mellitus (43.2%), chronic glomerulonephritis (25.1%), hypertension (8.3%), and chronic interstitial nephritis (2.8%) are major underlying comorbidities of ESRD^21^.

According to our results, the UTUC patients frequently suffered from pre-existing CKD and TI nephropathy than RCC ones, but UTUC ones with worse renal function had higher risk of abnormal GGS rate, and abnormal GGS Rate is histopathological predictor of creatinine doubling or dialysis within 5 years in not only UTUC patients but RCC ones. Because the renal histopathology of nephrectomized kidneys is a strong predictor, we suggest that pathophysiological analysis of nephrectomized kidneys can be performed to assess the renal outcomes of UTUC patients after unilateral nephrectomy.

### Limitation

The study has several limitations. First, this is a single institute study and selection biased cannot be preventable. A larger multi-institute may be needed to confirm our findings. Then, GGS and TI score are not accurate methods to evaluate chronic kidney injury. The scoring of tubulointerstitial injury is relative subjective, and GGS will be affected by the representativeness of the sampled tissue. Finally, the environmental factors that the patient exposed to are difficult to collect, such as the relationship between the UTUC patients and AA exposure.

## Conclusion

We confirmed the relevance of the renal histopathology of nephrectomized kidneys in predicting the renal survival rate of both UTUC and RCC patients receiving unilateral nephrectomy. Our findings indicated that UTUC patients had significantly higher risk of pre-existing CKD, abnormal GGS rate, and TI score than RCC ones, but UTUC with worse renal function had higher risk of abnormal GGS rate. Patients with hypertension, pre-existing CKD before unilateral nephrectomy and abnormal GGS rate in the renal histopathological analysis of nephrectomized kidneys from unilateral nephrectomy had a higher risk of creatinine doubling or developing ESRD within 5 years in not only UTUC but RCC patients. Thus, we may lend consideration to routine histologic evaluation of the non-tumorous kidney.

## Additional file

Additional file 1: **Table S1a.** Factors associated with pre-existing CKD by logistic regression in UTUC group. **Table S1b.** Logistic regression for Abnormal GGS rate in UTUC group. **Table S2a.** Factors associated with preexisting CKD by linear regression in RCC group. **Table S2b.** Factors associated with TI score by logistic regression in RCC group. (DOCX 55 kb)

## Abbreviations

AA: Aristolochic acid
AAN: Aristolochic acid nephropathy
ANOVA: One-way analysis of variance
CKD: Chronic kidney disease
CKD-EPI: CKD Epidemiology Collaboration Equation
eGFR: Estimated glomerular filtration rate
ESRD: End-stage renal disease
FFPE: Formalin-fixed paraffin embedded
GGS: Global glomerulosclerosis
H&E: Hematoxylin-eosin
HK-2: Human renal proximal tubule (cell line)
K-DOKI: Kidney Dialysis Outcomes Quality Initiative
NAD(P) H: Nicotinamide adenine dinucleotide phosphate
NQO1: NAD(P)H: quinone oxidoreductase 1
PAS: Periodic acid-Schiff
RCC: Renal cell carcinoma
SD: Standard deviation
TI: Tubulointerstitial
UC: Urothelial carcinoma
UTUC: UTUC, upper urinary tract urothelial carcinoma
